# Impacts of the DPP-4 Inhibitor Saxagliptin and SGLT-2 Inhibitor Dapagliflozin on the Gonads of Diabetic Mice

**DOI:** 10.3390/biomedicines11102674

**Published:** 2023-09-29

**Authors:** Ali A. Alshamrani, Mohammed A. Al-Hamamah, Norah A. Albekairi, Mohamed S. M. Attia, Sheikh F. Ahmad, Mohammed A. Assiri, Mushtaq A. Ansari, Ahmed Nadeem, Saleh A. Bakheet, Wael A. Alanazi, Sabry M. Attia

**Affiliations:** Department of Pharmacology and Toxicology, College of Pharmacy, King Saud University, Riyadh 11451, Saudi Arabia; aaalshamrani@ksu.edu.sa (A.A.A.); mohammad_9119@hotmail.com (M.A.A.-H.); nalbekairi@ksu.edu.sa (N.A.A.); mohamed.sabre2023@gmail.com (M.S.M.A.); fashaikh@ksu.edu.sa (S.F.A.); moassiri@ksu.edu.sa (M.A.A.); muansari@ksu.edu.sa (M.A.A.); anadeem@ksu.edu.sa (A.N.); sbakheet@ksu.edu.sa (S.A.B.); waalanazi@ksu.edu.sa (W.A.A.)

**Keywords:** hyperglycemia, chromosomal aberration, metabolism, reproduction, oxidative stress

## Abstract

Diabetes mellitus is a metabolic disease that can cause systemic problems, including testicular dysfunction. Several diabetes medications have demonstrated potential adverse effects on the male reproductive system; however, the effects of saxagliptin and dapagliflozin have not been sufficiently examined. This investigation studied the impacts of saxagliptin and dapagliflozin treatments on the gonads in a male mouse model of diabetes. Testicular disturbances were assessed by sperm DNA damage, diakinesis-metaphase I chromosome examination, and spermiogram analysis. Our results showed more sperm DNA damage, more spermatocyte chromosome aberrations, lower sperm motility/count, and more sperm morphological anomalies in diabetic mice than in the control mice. Dapagliflozin significantly restored all examined measures to the control values in diabetic mice, unlike saxagliptin, which exacerbated the reduction in sperm count and motility. Both drugs significantly restored the gonadal redox imbalances in diabetic mice by decreasing reactive oxygen species accumulation and increasing glutathione levels. In conclusion, our study presents preliminary evidence for the safety and efficacy of dapagliflozin in alleviating testicular abnormalities induced by diabetes, making it a promising candidate drug for patients with diabetes in their reproductive age. As saxagliptin may have negative effects on fertility, its prescription should be avoided in young male diabetic patients.

## 1. Introduction

The increasing frequency of diabetes mellitus in young men appears to be contributing to the global decrease in male fertility. Up to 40% of males with diabetes have coexisting gonadal problems, which supports the crucial link between metabolism and reproduction [1]. Although the mediators of this link are unknown, finding targeted therapeutics is essential for maintaining adequate glycemic control for the management of coexisting metabolic complications such as the impairment of male reproductive function [2]. Human reproduction requires the adequate maintenance and functioning of several organs and systems. Adequate testicular function that produces healthy sperm is critical for male reproduction; however, several other factors contribute to successful fertilization, including the maintenance of a subtle balance between estrogen and progesterone, successful spermatogenesis, the maintenance of suitable conditions for mature sperm storage, successful intercourse, and successful ejaculation [3]. All of these biological processes and organs can be affected in male dysmetabolic patients, which can have a negative effect on both reproductive function and pregnancy outcomes.

Glucose metabolism is a crucial component of spermatogenesis and the maintenance of basic activities, including motility and fertilization ability, in mature sperm. Male gonads are functionally distinguished by continuous sperm production with high-energy needs, and the significant metabolic activities of sperm cells make them particularly susceptible to any internal or external perturbation of the physiological milieu [2,4]. Both type I and type II diabetes cause testicular injury and male infertility by affecting hormones of the hypothalamic–pituitary–gonadal axis, resulting in testicular damage and altered sperm count and quality [2,4].

Several human and animal studies have demonstrated that uncontrolled diabetes causes a wide range of reproductive abnormalities, including low testosterone levels, impaired spermatogenesis, structural alterations in the testis, and decreased sperm count and motility [2,5,6]. In diabetic animal models, antidiabetic medications such as insulin and metformin have been shown to improve or restore male fertility. In contrast, other hypoglycemic medications may negatively affect male fertility [7,8]. Several new classes of medications have been developed for the treatment of hyperglycemia, including glucagon-like peptide I (GLP-I) analogs, sodium-glucose co-transporter 2 (SGLT-2) inhibitors, and dipeptidyl peptidase 4 (DPP-4) inhibitors [9]. DPP-4 inhibitors, commonly known as gliptins, prevent DPP-4 activity to inhibit the rapid breakdown of endogenously generated GLP-1, consequently promoting glucose regulation without generating hypoglycemia. Saxagliptin is currently the most commonly marketed DPP-4 inhibitor and is regularly indicated for patients with diabetes and poor glycemic control, along with diet and exercise. SGLT-2 inhibitors, also known as gliflozins, inhibit SGLT-2 protein activity in the kidneys. In addition to blood glucose management, dapagliflozin provides considerable cardiovascular benefits to individuals with hypoglycemia. As they facilitate the excretion of glucose under hyperglycemic conditions without influencing insulin secretion, these medications have a low risk of inducing hypoglycemia [10].

To date, few studies have assessed the possible effects of these new classes on male reproductive function, and the available findings are contradictory [8]. However, due to their modes of action, they are likely to have direct or indirect effects on the male reproductive system. Additionally, examining the effects of these newly developed substances in diabetes is essential to elucidate their possible disruptive effects on the reproductive system and to avoid the transfer of these effects via gametes during conception [5]. Therefore, we hypothesis that understanding the safety of these medications is important for identifying appropriate management strategies and lowering the economic burden of diabetes.

Streptozotocin-induced diabetes is commonly used in animal models to study the effects of diabetes on various organs such as testicular cells because changes in the male reproductive system are not caused by a direct effect of streptozotocin, but rather by a reduction in insulin [11]. Streptozotocin administration can induce type I and type II diabetes, depending on the dose regimen. However, the resulting hyperglycemia caused by streptozotocin can be similar to either type I or type II diabetes mellitus [12]. This investigation aimed to examine whether saxagliptin and dapagliflozin affect streptozotocin-induced testicular damage in a type 1 diabetic mouse model. We assessed testicular chromosomal DNA damage and semen quality based on a sperm comet test, spermatocyte diakinesis-metaphase I chromosome examination, and spermiogram analysis in diabetic and non-diabetic mice exposed to saxagliptin or dapagliflozin treatments. Additionally, we investigated the potential protective mechanisms of saxagliptin and dapagliflozin on the oxidant–antioxidant imbalance in hyperglycemic animals.

## 2. Materials and Methods

### 2.1. Mice

Male C57BL/6J mice aged 7–9 weeks and weighing 18–23 g were purchased from the Experimental Animal Care Center at King Saud University (Riyadh, Saudi Arabia). Our study protocol was in harmony with the NIH Guides for the Care and Use of Laboratory Animals and was approved by the Institutional Animal Care and Use Committee of King Saud University (KSU-SE-23-57). All mice were kept in well-ventilated mouse housing at 23 ± 2 °C and a humidity of 50%, with 12 h dark/light cycles and with food and water available *ad libitum*. During the experiment, the animals were acclimated in plastic cages (five per cage) inside a well-ventilated room. There were 10 mice in each treatment and control group.

### 2.2. Induction of Experimental Diabetes

Diabetes was caused by intraperitoneal administration of freshly prepared streptozotocin solution in 0.1 M citrated buffer, pH 4.5, at a dose of 60 mg/kg/d for 5 d consecutively [13]. To reduce the risk of early mortality, the mice were administered drinking water supplemented with 10% sucrose for 2 d starting on day 5. Random blood sugar was assessed in tail vein blood 7 d following streptozotocin administration; mice with blood sugar levels of 300 mg/dL or more were designated as diabetic mice and involved in the experiments (blood glucose levels were measured weekly thereafter using an Accu-Chek glucometer).

### 2.3. Treatment

Dapagliflozin (Selleck Chemicals, Stratech Scientific Ltd., Suffolk, UK) and saxagliptin (Bristol-Myers Squibb, Pennington, NJ, USA) were formulated by dissolving the pure substances with 0.5% sodium carboxymethyl cellulose (CMC) in millipore water using a magnetic stirrer before administration and were then administered by oral gavages immediately after formulation. Mice were divided into six groups of 10 mice each, as follows: Group 1, non-diabetic control mice; Group 2, non-diabetic mice treated with saxagliptin; Group 3, non-diabetic mice treated with dapagliflozin; Group 4, diabetic mice; Group 5, diabetic animals receiving saxagliptin; Group 6, diabetic animals receiving dapagliflozin. The vehicle (0.5% CMC) was administered in equal amounts to control (Group 1) and diabetic animals (Group 4) once daily for 35 d consecutively. In treated groups, animals were administered 10 mg/kg saxagliptin (Group 2 and Group 5) or dapagliflozin (Group 3 and Group 6) orally by gavage, once daily for 35 d consecutively. The dose and regimen of treatment were selected based on previously published studies [14,15,16]. A few mice per group that were injected with streptozotocin died within weeks after diabetic induction. Thus, the number of each treated and control group had six mice at the end of the experiment, which were used for statistical analysis. Non-diabetic C57BL/6J mice (Group 7) treated with cyclophosphamide (40 mg/kg; Sigma-Aldrich, St. Louis, MO, USA) were used as a positive control for genotoxicity [17,18]. Mice were euthanized under isoflurane inhalational anesthesia 24 h after the last dose of saxagliptin or dapagliflozin. The final weights of the animals were recorded and compared with the initial weights. The testes were removed, blotted, and weighed using an electronic balance, and the testis coefficient was calculated as (testis weight/body weight) × 100%. The *cauda epididymis* of each animal was dissected and sperm were teased in fetal calf serum as described previously [19,20].

### 2.4. Sperm Comet Test

DNA damage in sperm was measured using the comet test as described before [21], with a few modifications. Briefly, the sperm suspension (10,000 cells) was diluted with 0.5% low melting agarose and layered on a clean slide precoated with 1.5% regular agarose. An extra agarose coating was applied to the second layer. After solidification, the slides were lysed in a solution containing 1% Triton X. Following 1 h lysis at 4 °C, spermatozoa were decondensed by dipping the slides in 20 mM DDT solution on ice for half an hour, followed by 90 min at room temperature in 4 mM 3,5-diiodosalicylic acid lithium salt. Sperm DNA was unwound by soaking the slides in an electrophoresis solution for 20 min prior to electrophoresis. For 15 min, the slides were neutralized in a neutralization solution. The slides were drained and dehydrated in cooled absolute alcohol for 30 min before storage in a dry place until staining with ethidium bromide and scanned under a fluorescence microscopy (20× magnification) to score a minimum of 150 cells from each coded slide, randomly, using Comet Assay IV software [22]. The level of DNA fragmentations is expressed as a tail intensity in percentage.

### 2.5. Spermatocyte Chromosome Examination

The first meiotic chromosomal preparation of the collected testes were prepared using an air-drying approach, as described previously [23,24]. The testes were dissected and immersed for 20 min in an isotonic trisodium citrate solution (2.2%) at 25 °C. The tunica albuginea was isolated and the seminiferous tubules were sampled to form cell suspensions. After centrifuging the suspension for 5 min at 1000 rpm, the pellets were suspended in a hypotonic trisodium citrate (1.1%) for 20 min and centrifuged again. Pellets were resuspended in the fixative (Carnoy’s solution) and placed on a microscope slides to liberate the chromosomes. Following drying overnight, the coded slides were stained with (5%) Giemsa solution, and at least 100 well-spread diakinesis metaphase I cells per mouse were evaluated for abnormalities using a light microscope.

### 2.6. Spermiogram Examination

Spermatozoa motility and counts were assessed using a Neubauer hemocytometer under a light microscope, following the WHO guidelines for the study of human’s sperm, and two counts for each mouse were averaged [25]. Spermatozoa suspensions were dispersed on the hemocytometer for sperm motility testing, and the grade/percentage of spermatozoa motility was calculated. For sperm-shaped anomalies, a fraction of each spermatozoa suspension was diluted with an aqueous eosin Y (1%), and smears were performed on coded slides half an hour later. Slides were studied under light microscopy after drying overnight. Anomalies were classified as previously described [26]. A minimum of 500 sperm were assessed per mouse for morphological abnormalities such as triangular, hookless, amorphous heads, and banana-shaped tails, among other tail anomalies [17].

### 2.7. Evaluation of Oxidative Stress

To investigate the effects of saxagliptin and dapagliflozin on the oxidative stress induced by diabetes, testes and spermatozoa were sampled to evaluate reactive oxygen species (ROS) and reduced glutathione (GSH) levels. Testicular levels of GSH were determined using 5,5′-dithiobis(2-nitro-benzoic acid), as described before [27]. The GSH was evaluated using a freshly prepared GSH standard solution [28]. A portion of the sperm solution was centrifuged, and the pellet was re-suspended in 0.2 mL 1× PBS before being mixed with equal volume of 5 μM 2′,7′-dichloro-dihydro-fluorescein at 37 °C for half an hour in the dark to generate 2′,7′-dichlorofluorescein [29]. The intensity was measured using a FLUOstar OMEGA reader at 485/520 nm wavelengths.

### 2.8. Statistics

Data are shown as a mean with SD. Results were checked for homogeneity and normality before analysis using non-parametric analysis of variance (ANOVA) with a Tukey–Kramer post-hoc test or Mann–Whitney U test and Kruskal–Wallis with Dunn’s post-hoc test. Significance was established at *p* < 0.05.

## 3. Results

### 3.1. General Characteristics of the Mice

No substantial alterations in body weight between the control and experimental mice before the start of the experiment were observed. At the end of the experiment, no weight loss or symptoms of toxicity were found in animals that received saxagliptin or dapagliflozin. As expected, weight gain was observed in the non-diabetic control mice. After the seventh week, the diabetic mice had lower body weights than the normal mice, but the saxagliptin- and dapagliflozin-treated diabetic groups had normal body weights and were heavier than the untreated diabetic mice (Table 1). The diabetic mice had lower testes/body weight ratios than the non-diabetic control mice, suggesting that diabetes damages testicular tissues. This harmful effect was reduced after treatment with both drugs. Testis weight in the dapagliflozin-treated diabetic mice recovered to the control level; however, the testis weight in the saxagliptin-treated diabetic mice remained lower than that in the control non-diabetic mice. Testis weights in the saxagliptin-administered mice were not considerably different from those in the diabetes group (Table 1). Blood glucose levels in the diabetic animals were substantially higher than those in the control animals (*p* < 0.01). Both dapagliflozin and saxagliptin showed no effect on blood sugar in the control animals but reduced elevated blood sugar in the diabetic animals compared with those in the untreated diabetic animals.

### 3.2. Effects of Saxagliptin and Dapagliflozin on Diabetes-Caused Sperm DNA Damage

**Figure 1:**
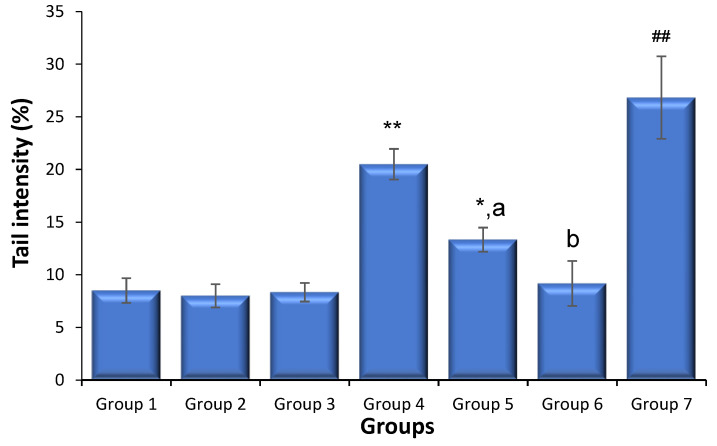
DNA damage in sperm cells of treated and untreated animals (mean ± SD, N = 6). Sperm cells were collected 24 h following the last dose of saxagliptin or dapagliflozin (10 mg/kg/d for 35 d) or cyclophosphamide. Group 1, non-diabetic control animals; Group 2, non-diabetic animals treated with saxagliptin; Group 3, non-diabetic animals treated with dapagliflozin; Group 4, diabetic mice; Group 5, diabetic animals treated with saxagliptin; Group 6, diabetic animals treated with dapagliflozin; Group 7, non-diabetic animals administered cyclophosphamide (40 mg/kg). * *p* < 0.05, ** *p* < 0.01 vs. control animals (Kruskal–Wallis test). ^a^ *p* < 0.05, ^b^ *p* < 0.01 vs. diabetic animals and ^##^ *p* < 0.01 vs. untreated non-diabetic animals (Mann–Whitney *U* Test).

Figure 1 shows the data of the sperm comet test. In the positive control-administered animals, sperm DNA breakage induced by cyclophosphamide administration was substantially greater than that in the control non-diabetic mice (*p* < 0.01). Both saxagliptin and dapagliflozin treatments had no significant influence on the frequency of sperm DNA strand breakage compared to that in non-diabetic animals. The diabetic animals exhibited a much greater percentage of sperm DNA strand breakage than the non-diabetic animals (*p* < 0.01). Nonetheless, when the diabetic animals were administered either saxagliptin or dapagliflozin, lower levels of sperm DNA fragmentations were detected compared to those in the diabetic animals (*p* < 0.01), with dapagliflozin providing the most effective reduction in the occurrence of DNA fragmentations.

### 3.3. Effects of Saxagliptin and Dapagliflozin on Diabetes-Caused Spermatocyte Chromosome Abnormalities

Table 2 shows the data of the spermatocyte diakinesis metaphase I examination. The number of induced abnormal primary spermatocytes in the positive control group was considerably greater than that in the control animals (*p* < 0.01). Neither saxagliptin nor dapagliflozin had an effect on the frequency of numerical and structural chromosome abnormalities compared to that in the non-diabetic control animals. Diabetes was found to increase the amount of total aberrant primary spermatocytes in the diabetic animals compared with that in the non-diabetic control mice (*p* < 0.01). As compared to the untreated diabetic animals, both saxagliptin and dapagliflozin therapy reduced the occurrence of abnormal primary spermatocytes caused by diabetes (*p* < 0.01). Furthermore, dapagliflozin induced an effect similar to that detected in the solvent-treated non-diabetic control animals.

### 3.4. Effects of Saxagliptin and Dapagliflozin on Diabetes-Caused Changes in Sperm Motility, Counts, and Abnormalities

As dapagliflozin treatment reduced sperm and testicular damage in diabetic mice, we aimed to determine whether saxagliptin and dapagliflozin treatment could improve the concentration and motility of sperm in diabetic mice. The spermiogram analysis (Table 3) showed that dapagliflozin administration to the non-diabetic animals had no influence on the sperm parameters compared to those of the untreated control animals. The diabetic animals had considerably lower sperm concentrations and motility than the non-diabetic animals (*p* < 0.01), wherein those were restored by dapagliflozin treatment (*p* < 0.01). In contrast, the saxagliptin-treated diabetic and non-diabetic animals showed a significant decline in sperm concentration and motility compared to the control non-diabetic group. The diabetic mice had considerably more sperm morphological abnormalities than the control animals (*p* < 0.01). Although the diabetic animals administered saxagliptin showed substantial improvement in sperm morphological abnormalities, the improvement did not match control levels. In contrast, dapagliflozin therapy successfully restored the sperm morphological abnormalities to normal levels in the diabetic mice (*p* < 0.01).

### 3.5. Effects of Saxagliptin and Dapagliflozin on Diabetes-Induced Oxidative Stress

**Figure 2:**
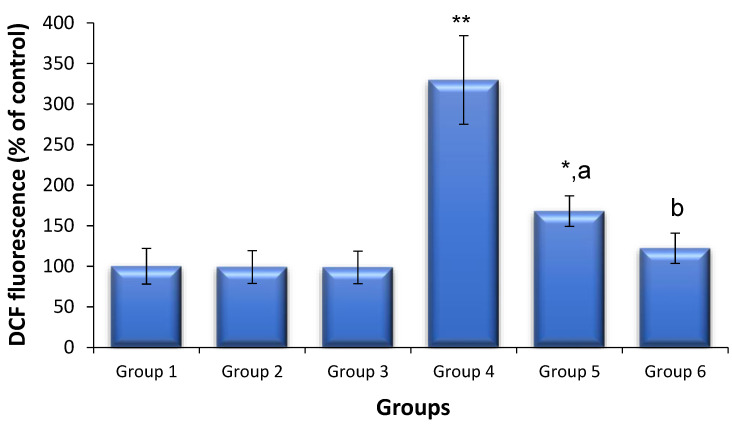
Sperm ROS levels in treated and untreated animals (mean ± SD, N = 6). Sperm cells were collected 24 h following the last dose of saxagliptin or dapagliflozin (10 mg/kg/d for 35 d). Group 1, non-diabetic control animals; Group 2, non-diabetic animals administered saxagliptin; Group 3, non-diabetic animals administered dapagliflozin; Group 4, diabetic animals; Group 5, diabetic animals administered saxagliptin; Group 6, diabetic animals administered dapagliflozin. * *p* < 0.05, ** *p* < 0.01 vs. control animals and ^a^ *p* < 0.05, ^b^ *p* < 0.01 vs. untreated diabetic animals (ANOVA test).

**Figure 3:**
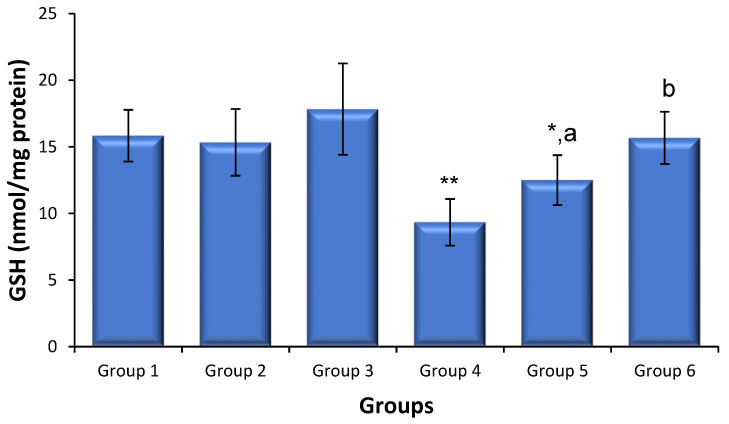
Sperm ROS and testicular GSH levels in treated and untreated animals (mean ± SD, N = 6). Sperm cells were collected 24 h following the last dose of saxagliptin or dapagliflozin (10 mg/kg/d for 35 d). Group 1, non-diabetic control animals; Group 2, non-diabetic animals administered saxagliptin; Group 3, non-diabetic animals administered dapagliflozin; Group 4, diabetic animals; Group 5, diabetic animals administered saxagliptin; Group 6, diabetic animals administered dapagliflozin. * *p* < 0.05, ** *p* < 0.01 vs. control animals and ^a^ *p* < 0.05, ^b^ *p* < 0.01 vs. untreated diabetic animals (ANOVA test).

ROS production and GSH levels were used to quantify the levels of oxidative stress (Figure 2). The level of sperm-generated ROS in the control mice did not differ following treatment with either saxagliptin or dapagliflozin compared with that in the untreated control mice. The levels of ROS production in diabetic mice were 3.2-fold those in the non-diabetic control animals (*p* < 0.01). However, saxagliptin and dapagliflozin therapy markedly reduced the diabetes-induced production of ROS to levels considerably lower than those in untreated diabetic animals. Testicular GSH levels did not differ between the mice treated with saxagliptin or dapagliflozin and the non-diabetic control animals. The amount of testicular GSH was lower in the diabetic mice than that in the non-diabetic control animals (*p* < 0.01); however, treatment with saxagliptin and dapagliflozin in the diabetic mice resulted in a significant recovery of GSH to control levels (Figure 3). Although saxagliptin therapy resulted in some GSH recovery in the diabetic animals compared with that in the untreated diabetic animals, the recovery was still significant when compared with the control group.

## 4. Discussion

Previous studies in animal models of streptozotocin-induced diabetes have revealed a variety of structural and functional male reproductive dysfunctions [2,11,30]. Consistent with previous findings, diabetic mice exhibited clear alterations in their testes, including a decreased testes/body weight ratio, increased sperm DNA fragmentations, increased spermatocyte chromosome aberrations, decreased sperm motility/count, and more spermatozoa morphological anomalies than those in non-diabetic control animals. The changes in these parameters indicate the major effect of diabetes induction on sperm maturation and development. In our study, dapagliflozin therapy completely reversed these alterations. In contrast, saxagliptin-treated diabetic animals had considerably lower sperm concentrations and motility than non-diabetic animals. Although diabetic animals administered saxagliptin recovered from DNA breaks and chromosomal abnormalities, the recovery did not match control levels.

Diabetes not only causes a variety of metabolic disorders but can also result in considerable chromosomal damage in reproductive cells. Several studies have demonstrated that chromosomal abnormalities in male and female gametes are the primary causes of early embryonic loss in humans [31,32,33]. There may be a threshold level for DNA breaking that results in an overload of the DNA damage/repair mechanism and an increase in abnormal metaphases. In our study, the administration of saxagliptin and dapagliflozin to non-diabetic mice did not cause additional spontaneous sperm DNA fragmentations or spermatocyte chromosome abnormalities, demonstrating the safety of the studied regimen in preventing testicular DNA damage. Diabetes increased the number of sperm DNA strand breakages and abnormal primary spermatocytes, which is consistent with previous studies [2,11,29,30,34]. However, when diabetic mice were administered saxagliptin and dapagliflozin, a substantial reduction in the level of sperm DNA strand breakage and abnormal primary spermatocytes was observed.

Diabetes impairs spermatogenesis and reduces sperm quality [32]. In the present investigation, decreased sperm count and motility were observed in diabetic mice, which were restored by the dapagliflozin treatment to the levels observed in the control animals. This relieving effect is consistent with the findings of Luo et al. [30], indicating that dapagliflozin may improve male reproductive potential, is a safe antidiabetic medication for young adults and adolescents, and is unlikely to impair reproductive function. In contrast, saxagliptin therapy was found to have a deleterious effect on sperm concentration and motility, independent of diabetes. Although improvements in other testicular and sperm parameters are assumed to be antioxidant-related, the decline in sperm concentration and motility indicates that the effect may be mediated by another mechanism.

Although research on DPP-4 inhibitors and male reproductive function is limited, the available data suggest that some drugs in these classes have an adverse impact on male fertility [35], warning against their use in men, especially during their reproductive years. Sperm production and differentiation are multistage processes involving several factors. DPP-4 is involved in spermatogenesis and has vital physiological functions in the male reproductive system [36]. The level of DPP-4 in the spermatozoa of patients with diabetes is much lower than that in normal healthy males, which could explain the loss of male fertility linked to diabetes [37].

A case study of a diabetic male who received the DPP-4 inhibitor sitagliptin revealed a progressive reduction in sperm volume, concentration, and motility, while the patient also had low free testosterone levels. Treatment was discontinued and the patient showed a subsequent recovery of semen quality [38]. Based on this study, DPP-4 inhibitors may impair fertility in men with diabetes mellitus. Sitagliptin therapy in streptozotocin-induced diabetic rats reduced the pathological alterations induced by diabetes in the weight and structure of male reproductive organs [39]. However, this medication did not completely reverse the diabetic phenotype. Furthermore, although the testes of sitagliptin-administered animals contained the greatest amount of spermatocytes at various mitotic phases, the medication failed to restore testosterone levels, while these rats also had lower levels of androgenic and estrogenic receptors in the seminal vesicles and epididymis [39].

Hyperglycemia promotes the development of highly reactive free radicals, which form AGEs with proteins involved in diabetic complication [40]. A decrease in antioxidant cellular defense systems in the gonads is associated with impairments of the primary reproductive organs owing to the glycation of scavenging enzymes [41,42]. Antioxidants may protect against diabetes-induced testicular oxidative damage and reproductive dysfunction by improving the gonadal antioxidant reserves and restoring disrupted endocrine systems [43]. Therefore, drugs with both antidiabetic and antioxidative properties can protect against both diabetes and its consequences. The formation of ROS was enhanced in diabetic animals compared to that in non-diabetic control animals, which was abolished by the injection of saxagliptin and dapagliflozin.

Reduced testicular GSH levels were also observed in diabetic animals, demonstrating impairment of the antioxidant defense mechanism(s). Because GSH could protect normal tissues from the deleterious impacts of ROS, the failure of antioxidant defense mechanisms can result in testicular dysfunction and increased chromosomal DNA damage [42]. In this study, GSH levels were restored in diabetic animals administered saxagliptin or dapagliflozin, which was in line with the antioxidant effects (lowering free radical levels and increasing antioxidant levels) previously observed in animals [30,44,45]. Thus, by rebalancing oxidant–antioxidant levels, saxagliptin and dapagliflozin could bolster cellular defenses against diabetes-related testicular problems.

Importantly, both drugs reduced oxidative stress in diabetic mice by improving antioxidant status, but they did not confer equivalent results in fertility. The reason for the discrepancy is not clear and requires future studies; however, it might be due to the differences in the mechanisms of action between saxagliptin and dapagliflozin. As mentioned above, the level of DPP-4 in the sperm cells of diabetic males is much lower than that of healthy males, and DPP-4 inhibitors may impair fertility in males with diabetes [38,39]. Thus, the differences in the mechanism of action between saxagliptin and dapagliflozin were reflected in their different effects on fertility in diabetic mice. The present study has several drawbacks, such as the use of a streptozotocin-induced type 1 diabetes model, which causes a decrease in endogenous insulin synthesis followed by the development of hyperglycemia and weight loss [46]. Additionally, this model is imperfect and exhibits certain other drawbacks, such as the fact that obesity is not its characteristic, as in type 2 diabetes. Thus, future detailed studies of diabetic transgenic animals and diabetic humans, as well as other parameters such as the relationship between diabetes and gut microbiome [47], might be interesting and might provide more information on the impacts of antidiabetic drugs on gonadal disturbances induced by hyperglycemia.

## 5. Conclusions

Persistent hyperglycemia is considered a key mechanism by which diabetes causes testicular impairment. This study presents a potential strategy for rescuing the adverse effects of diabetes, including reproductive dysfunction. The administration of saxagliptin and dapagliflozin to healthy mice had no notable adverse effects at the selected regimen. Dapagliflozin restored all examined parameters to their control levels in diabetic mice, whereas saxagliptin failed to do so and exacerbated the decrease in sperm count and motility. Both medicines reduced gonadal oxidative damage in diabetic mice by decreasing the formation of ROS and restoring lowered GSH levels. Our findings suggest that dapagliflozin can safely relieve gonadal abnormalities induced by diabetes and is a promising candidate drug for patients with diabetes in their reproductive age. Notably, saxagliptin may further impair male fertility and should thus be avoided in young male patients with diabetes.

## Figures and Tables

**Table 1:** Changes in body weight, testis weight, testis coefficient, and blood glucose level in treated and untreated animals (mean ± SD).

Groups	Body Weight (g)			Testis Weight (g)	Testis Coefficient (%)	Blood Glucose(mg/dL)
	Initial Weight	After Two Weeks	Final Weight			
Group 1	19.4 ± 1.5	23.3 ± 1.8	28.2 ± 1.4	0.198 ± 0.014	0.70 ± 0.02	145.6 ± 22.4
Group 2	20.3 ± 1.6	23.8 ± 1.4	27.3 ± 1.9	0.188 ± 0.011	0.70 ± 0.03	152.5 ± 24.7
Group 3	21.3 ± 1.7	22.8 ± 1.1	26.6 ± 1.8	0.190 ± 0.016	0.71 ± 0.04	149.1 ± 18.4
Group 4	20.8 ± 1.4	24.1 ± 1.2	21.5 ± 1.4 **	0.128 ± 0.011	0.59 ± 0.02 **	467.5 ± 39.3 **
Group 5	20.6 ± 1.5	24.2 ± 2.1	25.8 ± 1.7	0.168 ± 0.013	0.66 ± 0.01 *,^a^	231.8 ± 25.6 ^b^
Group 6	20.1 ± 1.8	22.5 ± 1.0	25.1 ± 1.4	0.176 ± 0.012	0.70 ± 0.03 ^b^	221.1 ± 22.5 ^b^

Group 1, non-diabetic control animals; Group 2, non-diabetic animals treated with saxagliptin; Group 3, non-diabetic animals treated with dapagliflozin; Group 4, diabetic mice; Group 5, diabetic animals treated with saxagliptin; Group 6, diabetic animals treated with dapagliflozin. * *p* < 0.05, ** *p* < 0.01 vs. control animals (Kruskal–Wallis test). ^a^ *p* < 0.05, ^b^ *p* < 0.01 vs. untreated diabetic animals (Mann–Whitney *U* Test).

**Table 2:** Frequency of spermatocyte diakinesis-metaphase I analysis in testes of treated and untreated animals (mean ± SD, N = 6).

Treatment Groups	Different Structural Chromosomal Aberrations Screened					Total Chromosomal Aberrations (%)
	X-Y Univalents	Autosomal Univalents	F/B	Polyploidy	MV	
Group 1	8	6	2	2	1	3.16 ± 0.98
Group 2	9	7	2	2	2	3.66 ± 1.21
Group 3	8	5	3	1	2	3.16 ± 0.98
Group 4	29	14	6	5	4	9.67 ± 1.94 **
Group 5	16	8	4	1	2	5.16 ± 0.98 ^a^
Group 6	13	4	4	1	1	3.83 ± 1.16 ^b^
Group 7	32	16	6	3	5	10.33 ± 2.58 ^##^

F, fragments; B, breaks; MV, multivalents having a chain of four chromosomes. Testes were collected 24 h following the last dose of saxagliptin or dapagliflozin (10 mg/kg/d for 35 d) or cyclophosphamide (40 mg/kg). Group 1, non-diabetic control animals; Group 2, non-diabetic animals treated with saxagliptin; Group 3, non-diabetic animals treated with dapagliflozin; Group 4, diabetic mice; Group 5, diabetic animals treated with saxagliptin; Group 6, diabetic animals treated with dapagliflozin; Group 7, non-diabetic animals treated with cyclophosphamide. ** *p* < 0.01 vs. control animals (Kruskal–Wallis test). ^a^ *p* < 0.05, ^b^ *p* < 0.01 vs. untreated diabetic animals and ^##^ *p* < 0.01 vs. untreated non-diabetic animals (Mann–Whitney *U* Test).

**Table 3:** Spermiogram examination of treated and untreated animals (mean ± SD, N = 6).

Treatment Groups	Mobility (%)			Morphology (%)			Sperm Counts (10^6^/^mL^)
	Fast	Slow	Immobile	Normal	Abnormal Heads	Abnormal Tails	
Group 1	58.5 ± 2.9	21.1 ± 1.2	20.3 ± 2.4	89.6 ± 1.5	5.5 ± 1.1	4.8 ± 0.5	57.1 ± 4.3
Group 2	48.5 ± 2.0 *	23.3 ± 1.8	28.1 ± 0.4 *	86.6 ± 1.2	6.8 ± 0.8	6.5 ± 0.8	51.3 ± 2.8 *
Group 3	56.1 ± 1.9	20.0 ± 2.5	23.8 ± 1.1	90.1 ± 1.3	5.3 ± 0.8	4.5 ± 0.5	54.8 ± 2.8
Group 4	40.3 ± 3.5 **	24.3 ± 2.1	35.3 ± 2.2 **	74.6 ± 1.7 **	10.8 ± 1.1 **	14.5 ± 1.1 **	40.3 ± 2.1 **
Group 5	37.5 ± 2.8 **	26.0 ± 2.1	36.5 ± 2.3 **	72.1 ± 2.7 **	13.1 ± 1.6 **	14.6 ± 1.3 **	33.3 ± 1.7 **
Group 6	54.0 ± 1.5 ^b^	21.8 ± 0.8	24.1 ± 1.4 ^b^	89.0 ± 1.0 ^a^	5.4 ± 0.5 ^b^	5.6 ± 0.6 ^b^	56.3 ± 2.2 ^b^

Sperm cells were collected 24 h following the last dose of saxagliptin or dapagliflozin (10 mg/kg/d for 35 d). Group 1, non-diabetic control animals; Group 2, non-diabetic animals administered saxagliptin; Group 3, non-diabetic animals administered dapagliflozin; Group 4, diabetic animals; Group 5, diabetic animals administered saxagliptin; Group 6, diabetic animals administered dapagliflozin. * *p* < 0.05, ** *p* < 0.01 vs. control animals and ^a^ *p* < 0.05, ^b^ *p* < 0.01 vs. untreated diabetic animals (ANOVA test).

## Data Availability

All data presented in this study are available on reasonable request from the corresponding author.
